# A Word about Communications

**Published:** 1870-10

**Authors:** 


### Editorial.

***Communications***

Are respectfully solicited from our subscribers in different sections of the country. It is a mistake to suppose that extraordinary cases are the only ones desired by readers. Sketches of the prevailing diseases, their general and local causes, and the modes of treatment found useful under all the circumstances, are matters which all thinking medical men are anxious to read. It is well known that we have great confidence in that large and intelligent class of the profession, an Eastern contemporary sneeringly denominates, "the country members." We wish to hear from them.

